# Enhanced Heterogeneous Graph Attention Network with a Novel Multilabel Focal Loss for Document-Level Relation Extraction

**DOI:** 10.3390/e26030210

**Published:** 2024-02-28

**Authors:** Yang Chen, Bowen Shi

**Affiliations:** 1 State Key Lab of Software Development Environment, Beihang University, Beijing 100191, China; 2 School of Journalism, Communication University of China, Beijing 100024, China; bowenshi@cuc.edu.cn

**Keywords:** relation extraction, heterogeneous graph neural network, entropy, attention mechanism, dependency tree

## Abstract

Recent years have seen a rise in interest in document-level relation extraction, which is defined as extracting all relations between entities in multiple sentences of a document. Typically, there are multiple mentions corresponding to a single entity in this context. Previous research predominantly employed a holistic representation for each entity to predict relations, but this approach often overlooks valuable information contained in fine-grained entity mentions. We contend that relation prediction and inference should be grounded in specific entity mentions rather than abstract entity concepts. To address this, our paper proposes a two-stage mention-level framework based on an enhanced heterogeneous graph attention network for document-level relation extraction. Our framework employs two different strategies to model intra-sentential and inter-sentential relations between fine-grained entity mentions, yielding local mention representations for intra-sentential relation prediction and global mention representations for inter-sentential relation prediction. For inter-sentential relation prediction and inference, we propose an enhanced heterogeneous graph attention network to better model the long-distance semantic relationships and design an entity-coreference path-based inference strategy to conduct relation inference. Moreover, we introduce a novel cross-entropy-based multilabel focal loss function to address the class imbalance problem and multilabel prediction simultaneously. Comprehensive experiments have been conducted to verify the effectiveness of our framework. Experimental results show that our approach significantly outperforms the existing methods.

## 1. Introduction

**Figure 1:**
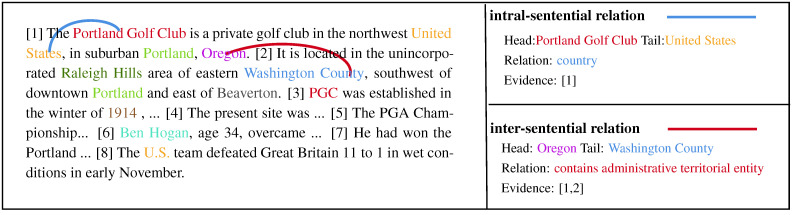
An example from dataset DocRED. Intra-sentential and inter-sentential are marked with blue and red lines, respectively [1,2,3,4,5,6,7,8].

Document-level relation extraction (DocRE) is defined as extracting all relationships between entities in multiple sentences of a document, which plays a critical role in many natural language processing tasks, such as knowledge graph construction, knowledge discovery, and knowledge-based question answering [1,2,3]. Compared to sentence-level relation extraction [4], DocRE is more challenging and harder to deal with for three main reasons: (1) Each entity in DocRE may have multiple mentions distributed across multiple sentences; (2) Relationships in the DocRE setting may span multiple sentences; (3) Logical reasoning is required to identify many complex relationships. Given these characteristics of DocRE, the relationships can be categorized into intra-sentential and inter-sentential relations. For instance, as shown in Figure 1, the entity mentions “*Portland Golf Club*” and “*United States*” are in the same sentence, so the relationship “*country*” between them is the intra-sentential relation. However, the entity mentions “*Oregon*” and “*Washington County*” span two sentences, and their relationship is an inter-sentential relation, which requires the model’s ability of non-local representations and relational inference.

Faced with these difficulties in DocRE, two primary categories of related works have emerged: sequence-based and graph-based methods. Sequence-based models [5,6,7] fall short in capturing the intricate structural information necessary for modeling long-distance inter-sentential relationships. Therefore, recent approaches predominantly rely on graph structures constructed from the document. These graphs offer improved modeling of structural information and long-distance dependencies. However, these graph-based works mostly focus on concept-level entity pairs to conduct relation prediction and inference, leading to a loss of context information contained in fine-grained entity mentions. We contend that relation prediction and inference should be grounded in specific entity mentions rather than abstract entity concepts. Furthermore, recent research [8] shows that there are totally three relation reasoning paths in the commonly used dataset for DocRE, which consists of an intra-sentential relation path and two inter-sentential relation paths (coreference reasoning path and logical inference path). These diverse relation path patterns pose a significant challenge to a model’s representational capacity and inference capabilities. To sum up, intra-sentential and inter-sentential relation extractions demand local mention-level representation and global mention-level representation, respectively, which is crucial for the model’s expressive capacity.

To address these challenges, we propose a two-stage mention-level framework based on an enhanced heterogeneous graph attention network for document-level relation extraction. To be concrete, firstly, we adopt a pre-coreference-resolution strategy to preprocess the input dataset. With the clues of evidence in the dataset, we can transform the coreference reasoning path into either an intra-sentential relation path or a logical inference path. Then, we construct a mention-aware heterogeneous graph from the input document based on the dependency tree. Entity mentions are connected to the nodes of the graph based on our designed rules. Secondly, we adopt two different strategies for intra-sentential and inter-sentential relations. For intra-sentential relation prediction, we directly utilize local representations to model relationships within a sentence. For inter-sentential relation, which involves relation inference, we employ a graph neural network to generate global representations for entity mentions. Unlike previous graph-based methods that mostly adopt graph convolutional neural-network-based models to model local and non-local representations, we design an efficient enhanced heterogeneous graph attention network. This network allows us to better capture long-distance semantic relationships.

Furthermore, for relational logical reasoning, we design an entity-coreference path-based inference strategy to conduct relational inference. Finally, in order to address the class imbalance problem and multilabel prediction simultaneously, inspired by circle loss [9] and focal loss [10], we introduce a cross-entropy-based multilabel focal loss function. To verify the effectiveness of our framework, extensive experiments have been conducted. Experimental results show that our approach significantly outperforms the existing methods. We summarize the main contributions of this paper as follows:

- We propose a two-stage mention-level framework for document-level relation extraction, which constructs a dependency-tree-based mention-aware heterogeneous graph and adopts different strategies for intra-sentential and inter-sentential relation prediction.
- For inter-sentential relation prediction and inference, we propose an enhanced heterogeneous graph attention network to better model long-distance semantic relationships and design an entity-coreference path-based inference strategy to conduct relation inference.
- We introduce a novel cross-entropy-based multilabel focal loss function to address the class imbalance problem and multilabel prediction simultaneously.
- Comprehensive experiments have been conducted to verify the effectiveness of our framework. Experimental results show that our approach significantly outperforms the existing methods.

## 2. Related Work

Early methods on relation extraction (RE) mainly concentrate efforts on sentence-level RE [4,11,12,13,14,15], which focuses on learning local context representation for relation predicting. These works achieve great success in this ideal scenario. However, there are numerous relational facts that can only be identified across sentences in the real scenario [7,16], which is extremely difficult for the sentence-level RE method to handle. Recent attempts have been made to deal with document-level relation extraction (DocRE) through various methods that can be generally categorized into sequence-based and graph-based methods.

Sequence-based models [5,6,7] encode the whole document to output each word’s context hidden representation, which is then used to predict the relation between entities. These models are not capable of fully capturing the structural information to model inter-sentential relationships over long distances. Therefore, graph-based methods [17,18,19] have sprung up recently. Christopoulou et al. [18] introduce a new edge-oriented graph neural model designed for document-level relation extraction employing multi-instance learning. Constructing a document-level graph featuring diverse types of nodes and edges, the authors model intra- and inter-sentence pairs concurrently using an iterative algorithm applied to the graph edges. Zeng et al. [20] propose a double-graph-based method that constructs a heterogeneous mention-level graph and an entity-level graph. Firstly, they introduce a heterogeneous mention-level graph (hMG) for the interaction among different mentions. Secondly, they present an entity-level graph (EG) and suggest an innovative path reasoning mechanism to facilitate relational reasoning among entities. However, their entity-level graph sacrifices some fine-grained information. Wang et al. [21] introduce rhetorical structure theory to select appropriate evidence and to reason relations. By incorporating rhetorical structure theory (RST) as external knowledge, they aim to select appropriate evidence and demonstrate the reasoning process on a novel document graph, RST-GRAPH. This graph indicates valid semantic associations between multiple text units through RST and integrates a set of reasoning modules to capture evidence efficiently. Zeng et al. [22] propose a separate architecture to represent intra-sentential and inter-sentential relations, respectively. Additionally, they present a novel logical reasoning module that models logical reasoning as self-attention among representations of all entity pairs. Xu et al. [8] propose a discriminative relation reasoning framework that uses three sub-tasks to model intra- and inter-sentence relations. Building upon this framework, they introduce a discriminative reasoning network (DRN). In this network, they utilize both the heterogeneous graph context and the document-level context to represent distinct reasoning paths. Tan et al. [23] propose a semi-supervised framework utilizing three components—axial attention, adaptive focal loss, and knowledge distillation module—to deal with different problems in DocRE. Firstly, an axial attention module is employed to enhance performance on two-hop relations by learning interdependencies among entity pairs. Secondly, an adaptive focal loss is introduced to address the class imbalance issue in DocRE. Lastly, knowledge distillation is utilized to reconcile differences between human-annotated data and distantly supervised data. Different from most previous works based on concept-level entity representations, our method proposes a two-stage framework to deal with intra- and inter-sentential relations based on mention-level representations.

## 3. Methodology

**Figure 2:**
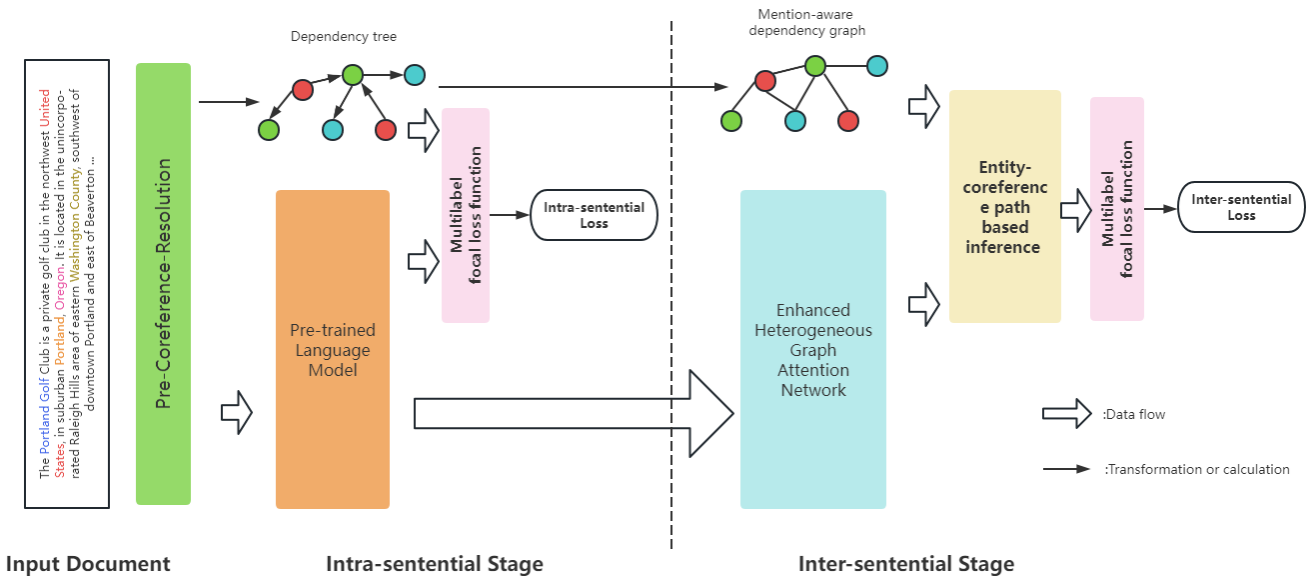
Overview of our two-stage framework for intra-sentential and inter-sentential relation extractions.

In this part, we introduce our two-stage mention-level framework based on enhanced heterogeneous graph attention network for document-level relation extraction. Figure 2 illustrates the overview of our method.

### 3.1. Task Formulation

Here, we first present the task formulation of document-level relation extraction. Given a document D consisting of *n* sentences $\left\{s_{0},s_{1},s_{2}\ldots s_{n-1}\right\}$ and a set of entities $E=\{e_{0},e_{1},e_{2}\ldots$$e_{m-1}\}$, each entity $e_{i}$ in E may have multiple mentions, denoted as ${\left\{m_{j}\right\}}_{0}^{k}$, distributed in different sentences of document D. For pairs of entities in E, the objective of document-level relation extraction (DocRE) is to predict relationship y∈R of the entity pairs, where R is the predefined relation set including “NA”, which means there is no relation between the given entity pair. Note that there may be multiple relations between the same entity pair.

### 3.2. Model Architecture

#### 3.2.1. Pre-Coreference-Resolution

**Figure 3:**
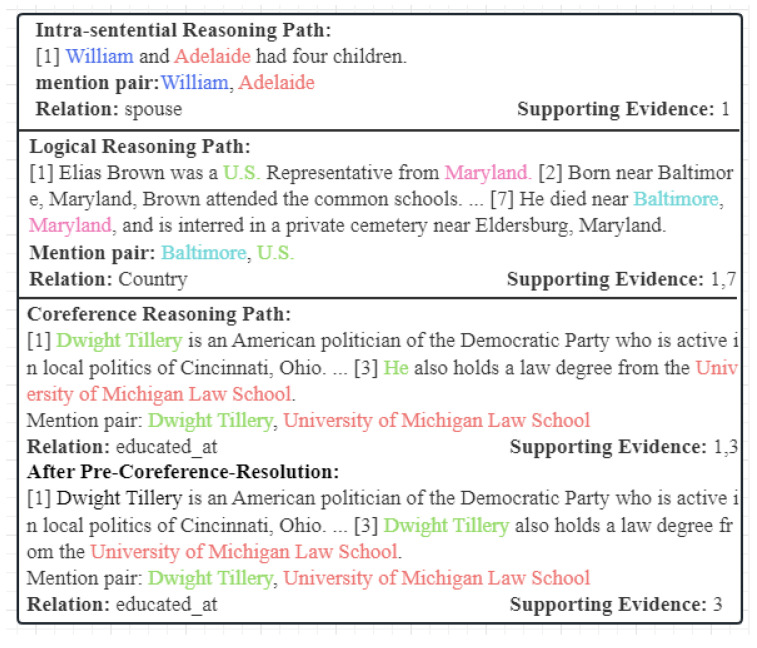
Examples of three relation reasoning paths and pre-coreference-resolution. Note that after pre-coreference-resolution, the coreference reasoning paths would be converted into intra-sentential relation paths [1,2,3,7].

Recent research [8] shows that there are totally three relation reasoning paths in the commonly used dataset for DocRE, which consists of an intra-sentential relation path and two inter-sentential relation paths. Furthermore, the two inter-sentential relation paths can be divided into the coreference reasoning path and the logical inference path. In our framework, we first use spaCy [24] to apply coreference resolution to the document of the dataset, whereby coreference reasoning paths would be converted to intra-sentential relation paths with the help of the annotated supporting evidence contained in the dataset. To better illustrate this process, we present corresponding examples for three relation reasoning paths and pre-coreference-resolution in Figure 3. For the intra-sentential relation path and the logical inference path, we will detail our methods for them in subsequent sections.

#### 3.2.2. Mention-Aware Dependency Graph Construction

**Figure 4:**
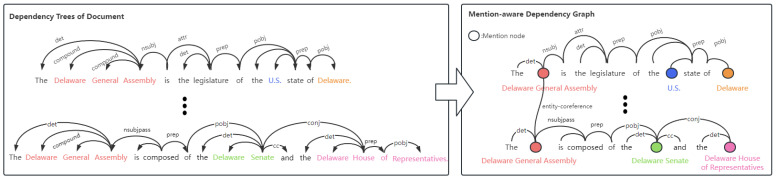
Illustration of construction procedure of mention-aware dependency graph. The circles represent the entity mention nodes in the graph. Mention nodes that refer to the same entity are connected with the “*entity-coreference*” edge.

In order to take advantage of the rich semantic information in the dependency tree, we construct our mention-aware dependency graph by designed rules. Specifically, we first use spaCy to transform each sentence of document D into a syntax dependency tree. Previous graph-based methods construct dependency trees based on fine-grained tokens, leading to the information loss of entity mention as a whole. Therefore, we replace the token nodes of entity mentions with corresponding mention nodes and, meanwhile, keep the fundamental semantic structure. Besides the semantic edges of the dependency tree, we connect the mention nodes, which belong to the same entity with a pre-defined edge type named “*entity-coreference*”, contributing to further relation inference. To illustrate clearly, we present the transformation in Figure 4.

#### 3.2.3. Context Encoder

We use pre-trained language models (PLMs) as our basic encoder to obtain context representations of the input document. Formally, the input document $D={\left\{w_{i}\right\}}_{0}^{n}$ is fed to the PLM to output the local context representations, which is formulated as

(1) $$H_{local}=PLM\left(D\right)$$

where $H_{local}$ is the output hidden vectors of the pre-trained language model.

#### 3.2.4. Enhanced Heterogeneous Graph Attention Network

To tackle the challenge of the heterogeneous dependency-based graph, previous works mostly adopt graph convolutional network-based models such as R-GCN [25], which is not efficient for relation inference due to its neglect of edge-type information. To model long-distance relationships, we propose an effective enhanced heterogeneous attention graph network, providing the global representations of entity mentions for inter-sentential relation inference.

**Attention-incorporated edge-type information.** Though R-GCN extends the graph convolutional network to a heterogeneous graph setting, it might not be optimal due to the equal importance of nodes from different edge types. Inspired by graph attention networks [26], we integrate edge-type embedding with an attention mechanism to enable the model to focus on crucial information from specific edge types. To be concrete, we embed each edge type into a d-dimensional vector. The attention can be formulated as follows:

(2) $$\alpha_{ij}=\frac{exp\left(LeakyReLU\left(a^{⊤}\left[Wh_{i}||Wh_{j}||R_{\phi \left(i,j\right)}\right]\right)\right)}{\sum_{k\in N_{i}}exp\left(LeakyReLU\left(a^{⊤}\left[Wh_{i}||Wh_{k}||R_{\phi (i,k)}\right]\right)\right)}$$

where $N_{i}$ is the neighbor set of node i, *W* is a learnable transformation matrix, and $R_{\phi (i,j)}$ refers to the edge-type embedding between node i and node j. During training, the edge-type embedding matrix *R* is learnable.

**Residual attention.** To make the graph network deeper, inspired by [27], we use pre-activation residual connection in node aggregation. In addition, RealFormer [28] shows that the residual attention layer is beneficial. So, we add residual connection to the attention layer. The above process can be formulated as follows:

(3) $$\begin{array}{c} h_{i}^{l}=\sigma \left(\sum_{j\in N_{i}}\alpha_{ij}^{l}W^{l}h_{j}^{l-1}+h_{i}^{l-1}\right) \end{array}$$

(4) $$\begin{array}{c} \alpha_{ij}^{l}=\frac{exp\left(att\_score^{l}\left(h_{i}^{l},h_{j}^{l}\right)+att\_score^{l-1}\left(h_{i}^{l-1},h_{j}^{l-1}\right)\right)}{\sum_{k\in N_{i}}exp\left(att\_score^{l}\left(h_{i}^{l},h_{k}^{l}\right)+att\_score^{l-1}\left(h_{i}^{l-1},h_{k}^{l-1}\right)\right)} \end{array}$$

(5) $$\begin{array}{c} att\_score^{l}\left(h_{i}^{l},h_{j}^{l}\right)=LeakyReLU\left(a^{⊤}\left[W^{l}h_{i}||W^{l}h_{j}||R_{\phi \left(i,j\right)}\right]\right) \end{array}$$

where σ is an activation function.

**Multihead attention.** To further boost the network’s expressive capacity, we use a multihead attention mechanism adopted by previous works. In this setting, the rules for updating node i can be formulated as:

(6) $$h_{i}^{l}=\sigma \left(|{|}_{k=1}^{q}\sum_{j\in N_{i}}\alpha_{ijk}^{l}W_{k}^{l}h_{j}^{l-1}+h_{i}^{l-1}\right)$$

where σ is an activation function, ∣∣ represents the concatenation operation of vectors, *q* is the number of heads, and $\alpha_{ijk}^{l}$ is calculated by (4).

#### 3.2.5. Relation Classification

**Intra-sentential relation.** For intra-sentential relation prediction, we directly use the entity mention’s local representation to identify the relation. Specifically, given an entity mention pair $\left\{m_{h},m_{t}\right\}$ within a sentence, each mention’s local context embedding is calculated as $h=\frac{1}{l}\sum_{i=start}^{start+l}h_{i}$, where start is the position mention beginning with, *l* is the length of the mention, and $h_{i}$ is the output hidden vector of the pre-trained language model in the i-th position. We first concatenate the entity-type embedding and local mention context embedding from the PLM such as roberta [29]. To incorporate semantic structure information within the sentence, we also use the lowest common ancestor of the two mention nodes in the dependency tree as additional structural information. We use a gated linear unit (GLU) to fuse the representations. Formally, the logits score of the mentions pair can be calculated as follows:

(7) $$\begin{array}{c} score=W_{o}\left(\left(W_{u}{\hat{h}}_{local}\right)⊙\sigma \left(W_{v}{\hat{h}}_{local}\right)\right) \end{array}$$

(8) $$\begin{array}{c} {\hat{h}}_{local}=h_{i}||h_{j}||a_{ij} \end{array}$$

where ∣∣ stands for the concatenation operation of vectors, ⊙ is the point-wise multiplication, σ is the sigmoid activation function, $a_{ij}$ is the hidden vector of the lowest common ancestor of the two mention nodes in the dependency tree, and $W_{u},W_{v}\in R^{s\times d}$, and $W_{o}\in R^{r\times s}$ are learnable parameters.

**Inter-sentential relation.** For inter-sentential relation prediction involving relation inference, the global representations of entity mentions are indispensable, which are obtained by our enhanced heterogeneous graph attention network (EHGAT). We argue that the inter-sentential logical reasoning path always appears in a specific sentence pattern. Therefore, we design an entity-coreference path-based inference strategy to capture composite relations. First, we give the definition of the compoundable mention pair of entity-coreference paths.

**Definition 1.**  *Given entity mentions $m_{1}$, $m_{2}$ within sentence $s_{1}$ and mentions $m_{3},m_{4}$ within sentence $s_{2}$, if $m_{2}$, $m_{3}$ refer to the same entity and $m_{1}$, $m_{4}$ refer to different entities, $m_{1}$ and $m_{4}$ are defined as the compoundable mention pair of the entity-coreference path.*

According to the above definition, we can ensure that if there is a relation $R_{1}$ between $m_{1}$ and $m_{2}$, a relation $R_{2}$ between $m_{3}$ and $m_{4}$, a predefined relation is likely existing between $m_{1}$ and $m_{4}$. For a document D, we pick out all compoundable mention pairs of coreference paths. For each compoundable mention pair $m_{s}$ and $m_{o}$, we use a gated linear unit (GLU) to fuse the representations, and the logits score between them is formulated as follows:

(9) $$\begin{array}{c} score=W_{o}\left(\left(W_{u}{\hat{h}}_{global}\right)⊙\sigma \left(W_{v}{\hat{h}}_{global}\right)\right) \end{array}$$

(10) $$\begin{array}{c} {\hat{h}}_{global}=h_{s}||h_{o} \end{array}$$

where ⊙ is the point-wise multiplication, $h_{s},h_{o}$ are the global context embeddings of the mention pair $\left\{m_{s},m_{o}\right\}$, σ is the sigmoid activation function, and $W_{u},W_{v}\in R^{s\times d}$, and $W_{o}\in R^{r\times s}$ are learnable parameters. Note that the parameters $W_{u},W_{v}$, and $W_{o}$ differ from those in the intra-sentential section.

#### 3.2.6. Multilabel Focal Loss Function

According to our analysis of the dataset for DocRE, there may be multiple relationships between the mention pairs. So, the mention-level relation prediction should be regarded as a multilabel classification problem. Furthermore, most of the mention pairs have the “NA” relations, which means that the negative instances are much more than the positive instances, leading to the class imbalance problem. To address the above challenges simultaneously, inspired by circle loss [9] and focal loss [10], we introduce a cross-entropy-based multilabel focal loss function. Specifically, we first introduce a threshold class denoted as “TH”. The logits scores of positive classes are expected to be higher than the logits score of the threshold class, and the logits scores of negative classes are expected to be lower than the logits score of the threshold class, which can be formulated as follows:

(11) $$\begin{array}{cc} \; & L_{pos}^{n}=\log\left(exp\left(S_{TH}^{n}\right)+\sum_{i\in \Omega_{neg}}exp\left(S_{i}^{n}\right)\right)+\\ \; & \log(exp\left(-S_{TH}^{n}\right)+\sum_{j\in \Omega_{pas}}exp\left(-S_{j}^{n}\right)) \end{array}$$

(12) $$L_{neg}^{k}=-\log\frac{exp(S_{TH}^{k})}{\sum_{i\in \Omega }exp\left(S_{i}^{k}\right)}$$

where $\Omega_{neg}$ represents the set of negative classes, $\Omega_{pos}$ represents the set of positive classes, and $S_{i}$ represents the logits score of specific relation class. Finally, the total loss of the dataset is calculated as:

(13) $$\begin{array}{c} L=\sum_{n\in R_{pos}}L_{pos}^{n}+\sum_{k\in R_{neg}}{\left(1-p_{k}\left(NA\right)\right)}^{\gamma }L_{neg}^{k} \end{array}$$

(14) $$\begin{array}{c} p_{k}\left(NA\right)=\frac{exp(S_{TH}^{k})}{\sum_{i\in \Omega }exp\left(S_{i}^{k}\right)} \end{array}$$

where $R_{pos}$ is the set of positive instances, $R_{neg}$ is the set of negative instances, γ is a hyperparameter, and p(NA) is the probability of the “NA” relationship. By the above focal loss style loss function, the imbalance class problem is alleviated, leading to an improvement in performance.

## 4. Experiments

This section presents the details of our experiments, including the datasets, settings, baselines, hyperparameters, and experimental results.

### 4.1. Dataset

We use DocRED [7], the widely used benchmark of document-level relation extraction, to evaluate our method. DocRED is the largest human-annotated dataset for document-level relation extraction first proposed by Yao et al. [7]. It is constructed from Wikipedia and Wikidata and contains over 5000 documents, 132,375 entities, 96 relation types, and 56,354 relational facts. Different from the sentence-level relation extraction dataset, more than 40.7% of the relational facts in DocRED are inter-sentential relations, which can only be extracted from multiple sentences. In addition, evidence of relational facts is available in DocRED, which is critical for our approach. Details on DocRED are presented in Table 1.

### 4.2. Baseline Methods

We use recent competitive models as baselines for comparison, including Coref [30], SSAN [31], GAIN [20], ATLOP [32], DocuNet [33], EIDER [34], SAIS [35], HAG [36], and AFLKD [23].

**Coref** [30] presents a language representation model named CorefBERT, strengthening the coreferential reasoning ability of the BERT model.

**SSAN** [31] formalizes entity structure for document-level relation extraction and effectively incorporates such structural priors into both contextual reasoning and structure reasoning of entities.

**GAIN** [20] constructs two levels of graph structures: mention-level graph and entity-level graph, based on which entity interactions and relational logical reasoning are modeled.

**ATLOP** [32] proposes adaptive thresholding and localized context pooling for document-level relation extraction.

**DocuNet** [33] formulates document-level relation extraction as a semantic segmentation task and introduces the document U-shaped network.

**EIDER** [34] proposes a three-stage DocRE framework. It comprises joint relation and evidence extraction, evidence-centered relation extraction, and fusion of extraction results, which take advantage of the evidence sentences.

**SAIS** [35] explicitly teaches the model to capture relevant contexts and entity types by supervising and augmenting intermediate steps and further boosts the performance through evidence-based data augmentation and ensemble inference while reducing the computational cost.

**HAG** [36] proposes a heterogeneous affinity graph inference network, which utilizes coref-aware relation modeling and a noise suppression mechanism to address the long-distance reasoning challenges in document-level RE.

**AFLKD** [23] utilizes an axial attention module for learning the inter-dependency among entity pairs. It also proposes an adaptive focal loss to tackle the class imbalance problem and adopts knowledge distillation to make use of distantly supervised data in DocRED.

### 4.3. Implementation Details

Our model is implemented with Pytorch [37] and the Transformer library of Huggingface [38]. We use the pre-trained language model $ROBERTA_{large}$ [29] as the encoder of our framework. During training, our model is optimized with AdamW [39] based on the mixed-precision mode. We stack six layers in the enhanced heterogeneous graph attention network. The hyperparameter γ of the multilabel focal loss is set to 2.

Following previous works [7,32,34], we adopt F1 and Ign F1 scores as the standard evaluation metrics. The difference between F1 and Ign F1 is that the shared relation facts in the training and dev/test sets are not included in the Ign F1 metric’s calculation. For a fair comparison, our baseline models are all based on $ROBERTA_{large}$ [29], which our TSFGAT adopts. The experiments are conducted five times with different random seeds, and the average scores are reported. We train and evaluate our TSFGAT on four Tesla V100 16 GB GPUs.

### 4.4. Experimental Result

We report the experimental result in Table 2. It shows that our model significantly outperforms the existing baselines. Specifically, our method achieves 68.14 Ign_F1 and 69.93 F1 score, which is a new state-of-the-art result on DocRED. It suggests that our approach can capture mention’s both local context representation for intra-sentential relation extraction and global context representation for inter-sentential relation extraction. It is worth noting that among the above baselines, our method is the only one that incorporates both the evidence information contained in DocRED and the syntactic structure information.

## 5. Analysis

In this section, we discuss the influence of each module on our method and provide details of the ablation experiments. Additionally, we conduct a case study to explain the inference capacity of our approach.

### 5.1. Ablation Study

**Effect of two-stage strategy.** In order to evaluate the effectiveness of our two-stage strategy, which predicts intra-sentential and inter-sentential relations separately, we conduct an ablation study on DocRED and report the result in the third row of Table 3. Specifically, in the “*w/o two-stage strategy*” model setting, we delete the intra-sentential stage, which means that the intra-sentential relations are also predicted with the global mention representation of the inter-sentential stage. The experimental result shows that when we adopt the one-stage strategy the Ign_F1 and F1 scores drop by 2.82 and 3.31, respectively, demonstrating the effectiveness of our designed two-stage framework. It suggests that intra- and inter-sentential relation extractions are required for distinctive local and non-local mention-level representations.

**Effect of pre-coreference-resolution.** To evaluate the effectiveness of pre-coreference-resolution, we conduct an ablation study on DocRED and report the result in the fourth row of Table 3. Specifically, in the “*w/o pre-coreference-resolution*” setting, we delete the process of pre-coreference-resolution. The result manifests that the Ign_F1 and F1 scores significantly drop by 26.46 and 26.23, respectively. It proves that our framework relies on the process of pre-coreference-resolution to handle the coreference reasoning path, which is illustrated in Section 3.2.1.

**Effect of enhanced heterogeneous graph attention network.** In order to prove the effectiveness of the proposed enhanced heterogeneous graph attention network (EHGAT) as a whole, we conduct an ablation study on DocRED and report the result in the fifth row of Table 3. Specifically, in the “*w/o enhanced heterogeneous graph attention network*” setting, similar to earlier efforts, we utilize R-GCN [25] in the inter-sentential stage. The experimental result shows that the Ign_F1 and F1 scores drop by 1.85 and 2.21, respectively. It demonstrates the superiority of our proposed enhanced heterogeneous graph attention network compared to the widely used R-GCN model in previous works.

**Effect of attention with edge-type information.** To analyze the role of attention with edge-type embedding in our proposed EHGAT, we conduct an ablation study on DocRED and report the result in Table 3. Specifically, in the “*w/o attention with edge-type information*” setting, we only use node information to calculate the attention instead. The result shows that when we abandon type information in the attention calculation of the heterogeneous graph, the Ign_F1 and F1 scores drop by 0.52 and 0.92, respectively. It suggests that incorporating type information is beneficial for the modeling of heterogeneous graphs.

**Effect of residual attention.** To analyze the effect of residual attention in our proposed EHGAT, we conduct an ablation study on DocRED and report the result in Table 3. Specifically, in the “*w/o residual attention*” setting, we use vanilla attention without residual connection. The result shows that compared to the full model, the Ign_F1 and F1 scores drop by 1.01 and 1.17, respectively. It proves that the residual attention mechanism is effective for our multilayer heterogeneous graph network.

**Effect of multilabel focal loss.** In order to assess the effect of the proposed multilabel focal loss, we conduct an ablation study on DocRED and report the result in Table 3. To be concrete, in the “*w/o multilabel focal loss*” setting, we adopt the vanilla binary cross-entropy loss instead, which is adopted by most previous works. The result shows that compared to the full model, the Ign_F1 and F1 scores drop by 3.77 and 4.29, respectively. It demonstrates the effectiveness of our proposed multilabel focal loss for the imbalanced multilabel dataset.

### 5.2. Case Study

**Figure 5:**
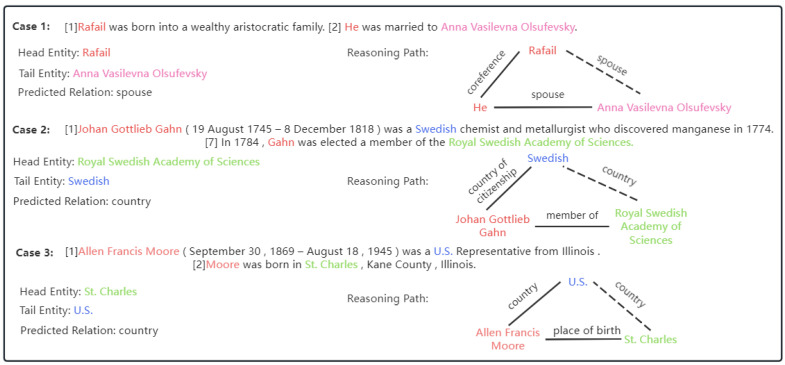
Three cases for illustrating the predictions of our TSFGAT. Case 1 involves the coreference reasoning path. Cases 2 and 3 involve the relational logical inference path [1,2,7].

To better show the inference capacity of our approach, we present several prediction cases of the development dataset of DocRED in Figure 5. To be concrete, Case 1 involves the coreference reasoning path. This case is converted to intra-sentential relation prediction by our Pre-Coreference-Resolution process. It shows our method’s ability to deal with the coreference reasoning path. As shown in Figure 5, Case 2 involves a relational logical inference path. If we want to determine the relationship “*country*” between entity mention “*Royal Swedish Academy of Sciences*” and “*Swedish*”, the model should capture the logical inference path: (*Johan Gottlieb Gahn, country of citizenship, Swedish*) + (*Gahn, member of, Royal Swedish Academy of Sciences*) = (*Royal Swedish Academy of Sciences, country, Swedish*). By our entity-coreference path-based inference strategy and enhanced heterogeneous attention networks, our model attains the global representations of mention “*Royal Swedish Academy of Sciences*” and “*Swedish*”, based on which relation inference is conducted to get the right answer “*country*”. Case 3 is similar to Case 2 in Figure 5, and we do not elaborate on it. The above cases show our model’s capacity for relation prediction and inference that is explainable.

## 6. Conclusions

In this paper, we propose a two-stage mention-level framework for document-level relation extraction, which constructs a dependency tree-based mention-aware heterogeneous graph and adopts different strategies for intra-sentential and inter-sentential relation prediction. For inter-sentential relation prediction and inference, we propose an enhanced heterogeneous graph attention network to better model the long-distance semantic relationships and design an entity-coreference path-based inference strategy to conduct relation inference. Furthermore, we introduce a cross-entropy-based multilabel focal loss function to address the class imbalance problem and multilabel prediction simultaneously. A series of experiments are conducted on the widely used benchmark of DocRE. Experimental results manifest that our approach significantly outperforms the existing methods. Further ablation analysis demonstrates the effectiveness of each component of our framework.

## Figures and Tables

**Table 1:** Statistics of benchmark DocRED.

Statistics	DocRED
# Train docs.	3053
# Dev docs.	1000
# Test docs.	1000
# Distant docs.	101,873
# Relations	97
Avg. # entities per doc.	19.5
Avg. # mentions per entity	1.4
Avg. # relations per doc.	12.5

**Table 2:** Experimental results of F1 and Ign F1 scores (%) on the DocRED dataset. In bold are the highest results.

Model	Evidence Information	Syntactic Structure	Dev		Test	
			Ign_F1	F1	Ign_F1	F1
Coref [30]	**✗**	**✗**	57.35	59.43	57.9	60.25
SSAN [31]	✔	**✗**	59.40	61.42	60.25	62.08
GAIN [20]	**✗**	**✗**	60.87	63.09	60.31	62.76
HAG [36]	**✗**	**✗**	60.85	63.06	60.78	60.82
ATLOP [32]	**✗**	**✗**	61.32	63.18	61.39	63.40
DocuNet [33]	**✗**	**✗**	62.23	64.12	62.39	64.55
EIDER [34]	✔	**✗**	62.34	64.27	62.85	64.79
SAIS [35]	**✗**	**✗**	62.23	65.17	63.44	65.11
AFLKD [23]	**✗**	**✗**	65.27	67.12	65.24	67.28
TSFGAT(ours)	✔	✔	**67.57**	**69.87**	**68.14**	**69.93**

**Table 3:** Ablation study on DocRED. Ign_F1 and F1 on test set are reported.

Model	Ign_F1	F1
Full model	68.14	69.93
w/o two-stage strategy	65.32	66.80
w/o pre-coreference-resolution	41.68	43.65
w/o enhanced heterogeneous graph attention network	66.29	67.72
w/o attention with edge-type information	67.62	69.01
w/o residual attention	67.13	68.76
w/o multilabel focal loss	64.37	65.64

## Data Availability

Data available on request due to restrictions, e.g., privacy or ethical. The data presented in this study are available on request from the corresponding author.
